# Pollen differentiation as well as pollen tube guidance and discharge are independent of the presence of gametes

**DOI:** 10.1242/dev.152645

**Published:** 2018-01-01

**Authors:** Barbara Glöckle, Wojciech J. Urban, Shiori Nagahara, Ellen D. Andersen, Tetsuya Higashiyama, Paul E. Grini, Arp Schnittger

**Affiliations:** 1 Department of Molecular Mechanisms of Phenotypic Plasticity, Institut de Biologie Moléculaire des Plantes du CNRS, IBMP-CNRS - UPR2357, Université de Strasbourg, 12 Rue du Général Zimmer, 67084 Strasbourg Cedex, France; 2 Section for Genetics and Evolutionary Biology, Department of Biosciences, University of Oslo, 0316 Oslo, Norway; 3 University of Hamburg, Biozentrum Klein Flottbek, Department of Developmental Biology, 22609 Hamburg, Germany; 4 Institute of Transformative Bio-Molecules (WPI-ITbM), Nagoya University, Furo-cho, Chikusa-ku, Nagoya, Aichi 464-8601, Japan; 5 JST ERATO Higashiyama Live-Holonics Project, Nagoya University, Furo-cho, Chikusa-ku, Nagoya, Aichi 464-8602, Japan

**Keywords:** Arabidopsis, Gametophyte, Fertilization, Chromatin, Transposons

## Abstract

After meiosis, an unequal cell division generates the male gamete lineage in flowering plants. The generative cell will undergo a second division, giving rise to the two gametes, i.e. the sperm cells. The other cell will develop into the vegetative cell that plays a crucial role in pollen tube formation and sperm delivery. Recently, the vegetative cell has been suggested to be important for programming of the chromatin state in sperm cells and/or the resulting fertilization products. Blocking the initial unequal division genetically, we first highlight that the default differentiation state after male meiosis is a vegetative fate, which is consistent with earlier work. We find that uni-nucleated mutant microspores differentiated as wild-type vegetative cells, including chromatin remodeling and the transcriptional activation of transposable elements. Moreover, live-cell imaging revealed that this vegetative cell is sufficient for the correct guidance of the pollen tube to the female gametes. Hence, we conclude that vegetative cell differentiation and function does not depend on the formation or presence of the actual gametes but rather on external signals or a cell-autonomous pace keeper.

## Abstract (precis)

**Summary:** Cell biological analyses in *Arabidopsis* show that the vegetative cell differentiates without the presence of the actual gametes, and is solely sufficient for pollen tube germination, guidance, ovule penetration and pollen tube discharge.

## INTRODUCTION

In contrast to animals, plants do not generate gametes directly through meiosis. Instead, meiosis gives rise to spores, so-called megaspores on the female and microspores on the male side, that have half the DNA content of the parent. From these spores, two sex-specific, typically haploid organisms are produced, the gametophytes, that undergo a distinct series of cell divisions generating the gametes.

In flowering plants, two gametes produced by each sex fuse during double fertilization. The male gametes, i.e. sperm, are produced from microspores by two mitotic divisions, designated pollen mitosis I (PMI) and pollen mitosis II (PMII) (McCormick, 2004; Borg et al., 2009; Twell, 2011). PMI is an unequal cell division that produces two cells with strikingly different fates. The so-called generative cell will continue with PMII and give rise to the actual gametes. The other cell is the vegetative cell, which will exit the cell cycle.

Given that the sperm cells are contained within the vegetative cell of a pollen grain, it is an unresolved issue to what degree an interaction between the individual pollen cells is required for their differentiation. Evidence for communication between pollen cells came from the observation that the sperm in tobacco and other species are connected by a cytoplasmatic bridge (Yu and Russell, 1993). Moreover, small RNAs that presumably originate from the vegetative cell can be detected in sperm cells of *Arabidopsis* (Slotkin et al., 2009).

Furthermore, to what degree the different cells in the pollen collaborate and/or individually contribute to pollen functions such as pollen tube growth and guidance is largely undetermined. There is ample evidence that the vegetative cell plays a key role in pollen tube growth (Higashiyama and Takeuchi, 2015), as exemplified by studies of the *CELLULOSE SYNTHASE-LIKE D4* gene (*CSLD4*), which is strongly expressed in vegetative cells and required for proper pollen tube growth (Bernal et al., 2008; Wang et al., 2011).

To what extent sperm cells contribute to pollen tube guidance is less clear. Previously, *HAPLESS 2* (*HAP2*), also known as *GENERATIVE CELL-SPECIFIC 1* (*GCS1*), which is expressed only in sperm, was reported to be required for efficient pollen tube guidance to ovules (von Besser et al., 2006). However, combining *gcs1/hap2* with the *quartet* (*qrt*) mutant, in which the male meiotic products stay together as one unit, Takahashi et al. reported recently that all four pollen tubes generated from *qrt* mutant pollen, i.e. the two wild-type like and the two mutant for *gcs1/hap2*, target ovules normally (Takahashi et al., 2017). Furthermore, many other germline mutants in which the formation and/or differentiation of sperm is compromised and results in fertilization defects, such as *duo pollen 1* (*duo1*), *duo3* and *duo1-activated zinc finger 1* (*daz1*) *daz2* double mutants, are successfully transported and discharged into the embryo sac (Rotman et al., 2005; Brownfield et al., 2009; Borg et al., 2014).

To untangle the function of individual pollen cells, interfering with cell division control is a promising approach, as a failure to progress through one or more pollen mitoses leads to pollen with fewer cells. Using colchicine, which compromises the mitotic spindle, Eady et al. could show that a unicellular microspore expressed a vegetative cell marker and could even produce a pollen tube (Eady et al., 1995). Similarly, the single-celled pollen of mutants in which pollen cytokinesis is affected, such as *gemini pollen 1* (*gem1*), expressed a vegetative cell fate reporter, indicating that cell division is not required for cell fate acquisition (Park et al., 1998).

Subsequent analyses of cell cycle regulator mutants in particular has allowed the further dissection of pollen development (Borg et al., 2009). This is exemplified by mutants in the cell cycle regulator, e.g. *cdka;1*, that result in pollen grains with a vegetative cell and only one sperm cell (Iwakawa et al., 2006; Nowack et al., 2006). Lack of PMII apparently does not interfere with the differentiation of a generative cell into a cell that can carry out sperm function, as this cell could fertilize one of the female gametes (Nowack et al., 2006; Aw et al., 2010). Similarly, expression of the translational inhibitor diphtheria toxin A from the *HAP2* promoter blocked PMII, resulting in pollen with a vegetative cell and only a single sperm-like cell that was capable of fertilizing one of the two female gametes (Frank and Johnson, 2009).

Mutants in the F-box protein, which is encoded by the *FBL17* gene, also produced single sperm pollen, likely through a pathway that controls CDKA;1 activity (Kim et al., 2008; Gusti et al., 2009; Zhao et al., 2012). This pathway involves the upstream-acting transcription factor E2F, which is well known to control entry into the DNA replication phase of the cell cycle in animals (Dick and Rubin, 2013). Similarly in plants, E2F is kept in an inactive state by a retinoblastoma protein called RETINOBLASTOMA RELATED 1 (RBR1) in *Arabidopsis* (Sabelli and Larkins, 2009; Gutzat et al., 2012; Desvoyes et al., 2014; Harashima and Sugimoto, 2016). A major target of E2F is FBL17, which mediates the degradation of CDKA;1 inhibitors of the KIP-RELATED PROTEIN (KRP) class as part of the SKIP-CULLIN-F-BOX (SCF) complex (Kim et al., 2008; Gusti et al., 2009; Zhao et al., 2012; Noir et al., 2015). Hence, loss of *FBL17* results in higher KRP levels and subsequently lower CDKA;1 activity.

Interestingly, the simultaneous loss of *CDKA;1* and *FBL17* gives rise to plants that produce single-celled pollen at anthesis (Zhao et al., 2012). Similarly, loss of E2F activity in combination with *fbl17* mutants also results in single cell pollen (Zhao et al., 2012). These mutants set the stage for a genetics approach for investigating the interaction of pollen cells that is presented in this report.

## RESULTS AND DISCUSSION

### Single-celled *cdka;1 fbl17* mutant pollen expresses vegetative but not generative/sperm cell fate markers

Consistent with previous analyses (Zhao et al., 2012), we found that the double heterozygous mutant *cdka;1^+/−^ fbl17^+/−^* produced ∼10% pollen at anthesis with only a single cell next to ∼50% tri-cellular (wild-type like) and 40% pollen with two cells (Fig. S1). The reduction in the number of pollen cells results from delayed/failed PMI and PMII of *cdka;1* and *fbl17* mutant microspore/pollen at the time point when CDKA;1 protein levels carried over from somatic cells fall and the accumulation of CDK inhibitor proteins starts. This is due to the reduced degradation of these inhibitors in the absence of FBL17 (Nowack et al., 2006; Kim et al., 2008; Zhao et al., 2012). In the following, we will refer to the mutant pollen with reduced cell numbers at anthesis as single-celled or two-celled pollen while earlier developmental stages before the first and second mitotic divisions in the wild type are referenced as uni-cellular microspores and bi-cellular pollen, respectively.

**Fig. 1.**
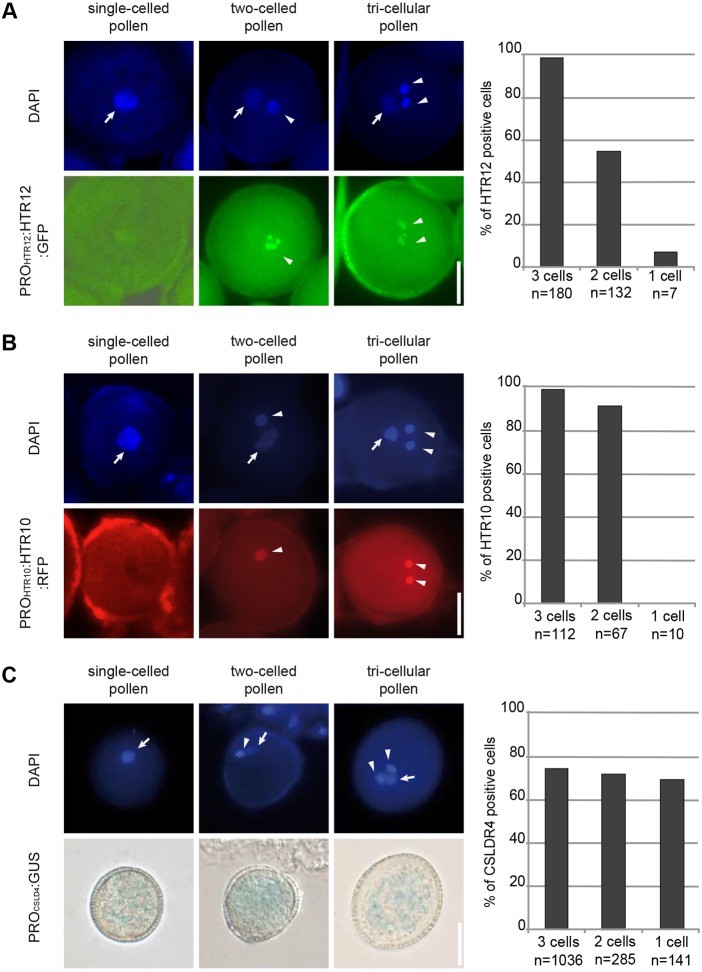
**Cell identity of single-celled pollen.** (A) Fluorescent micrographs of pollen containing a *PRO_HTR12_HTR12:GFP* sperm cell reporter and quantification. Scale bar: 10 µm. (B) Fluorescent micrographs of pollen containing a *PRO_HTR10_HTR10:RFP* sperm cell reporter and quantification. Scale bar: 10 µm. (C) Fluorescent and light micrographs of pollen containing a *PRO_CSLD4_GUS* vegetative cell reporter and quantification. Arrowheads indicate sperm cell nuclei; arrows indicate vegetative nuclei. Scale bar: 5 µm.

To reveal the developmental fate of single-celled pollen, we first introgressed two reporters for generative/sperm cell fate into *cdka;1^+/−^ fbl17^+/−^* mutants. To achieve this, we used two histone reporter lines, *HTR10* [also called *MALE-GAMETE-SPECIFIC HISTONE H3* (*MGH3*)] and *HRT12* [also called *CENTROMERIC HISTONE H3* (*CENH3*)], which have been found to accumulate in the nucleus of the generative but not in the vegetative cell during pollen maturation (Fang and Spector, 2005; Okada et al., 2005; Ingouff et al., 2007; Aw et al., 2010) (Fig. S2A). Analysis of RFP (for *PRO_HTR10_:HTR10:RFP*) and GFP (for *PRO_HTR12_:HTR12:GFP*) fluorescence after staining the nuclear DNA with 4′,6-diamidino-2-phenylindole (DAPI) showed that single-celled pollen did not express these markers (Fig. 1A,B). Notably, *HTR12* accumulates in unicellular microspores in the wild type (Chen et al., 2009; Ravi et al., 2011). Here, we found that HTR12 is also present in the unicellular microspores of *cdka;1^+/−^ fbl17^+/−^* mutants that are released after meiosis, indicating that the single-celled pollen of *cdka;1^+/−^ fbl17^+/−^* mutants at anthesis is not just a developmentally delayed/arrested microspore (Fig. S3). To further substantiate our observation, we scanned through several *cdka;1^+/−^ fbl17^+/−^* plants and found that out of 35 cases of single-celled pollen, not one expressed HTR12.

To complement these studies, we sought a reporter that indicates vegetative cell fate. To achieve this, we generated a promoter reporter line for *CSLD4*, which has previously been found to be strongly expressed in vegetative cells after completion of PMI (Bernal et al., 2008; Wang et al., 2011). Consistent with earlier analyses (Bernal et al., 2008; Wang et al., 2011), plants that express *β-glucuronidase* (*GUS*) behind a 5′ fragment of the *CSLD4* gene, showed strong blue precipitates in their pollen after incubation with the GUS substrate X-Gluc (Figs S2B and S4). When this line was introgressed to *cdka;1^+/−^ fbl17^+/−^* mutants, the single-celled pollen also showed strong GUS staining, consistent with a vegetative fate of the single cell (Fig. 1C). These results are in accordance with earlier work in which a chemically induced block of PMI gave rise to single-celled pollen grains in which the tomato-derived and vegetative-cell specific *LAT52* promoter is active (Twell, 1992; Eady et al., 1995).

### Single-celled pollen shows the same chromatin dynamics as the vegetative cell of wild-type pollen

**Fig. 2.**
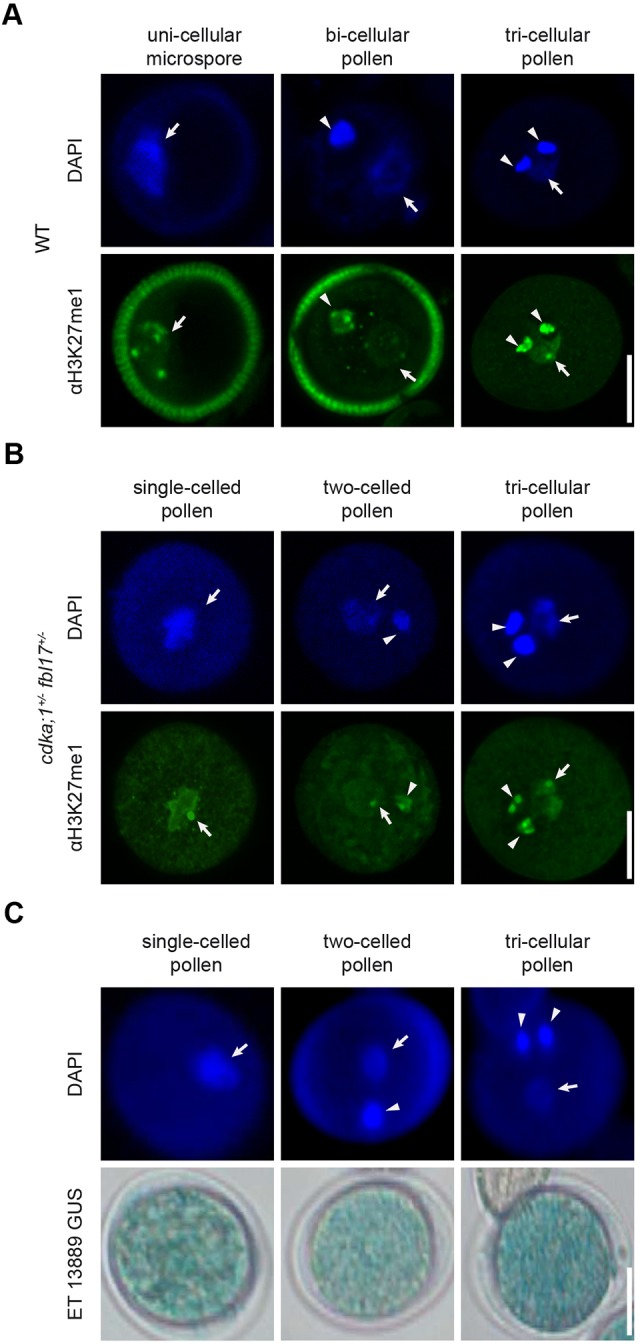
**Differentiation of chromatin in single-celled pollen.** (A) Fluorescent micrographs of wild-type pollen at different developmental stages stained with an antibody against H3K27me1 as a hallmark of heterochromatin. H3K27me1 becomes reduced in the vegetative cell of bi-cellular pollen and is almost absent in tri-cellular pollen except for one characteristic bright spot. Scale bar: 5 µm. (B) Fluorescent micrographs of mature pollen from *cdka;1^+/−^ fbl17^+/−^* plants stained with an antibody against H3K27me1. The H3K27me1 pattern in single-cell pollen nuclei resembles the vegetative cell nucleus of tri-cellular pollen with a single fluorescent spot. Scale bar: 5 µm. (C) Fluorescent and light micrographs of single-celled microspores, and two-celled and tri-cellular pollen of *cdka;1^+/−^ fbl/17^+/−^* plants expressing the Ds transposon enhancer trap line ET13889. Arrowheads indicate sperm cells; arrows indicate vegetative nuclei. Scale bar: 10 µm.

Recent work has shown that the vegetative cell undergoes substantial chromatin remodeling that can be revealed by histochemical investigation of mono-methylated lysine 27 on histone H3 (H3K27me1), a hallmark of heterochromatin (Jacob and Michaels, 2009; Slotkin et al., 2009; Schoft et al., 2011). In the uni-cellular microspore, H3K27me1 is predominantly found at chromocenters, which are highly compacted heterochromatic domains, including the pericentromeric regions (Fig. S2A). Hence, there are typically five brightly stained dots visible by immunocytological detection of this chromatin mark resembling the usual pattern of heterochromatin found in nuclei of many somatic cells of *Arabidopsis* (Fig. 2A) (Schoft et al., 2011). After the first mitotic division of the microspore, this pattern is present in both the nuclei of the generative and the vegetative cell (Fig. 2A). However, after the second mitotic division, the chromatin of the vegetative cell becomes restructured as visualized by an increase in space occupied by the nuclear DNA and a re-localization of H3K27me1 to a single spot (Fig. 2A). In contrast, the nuclear DNA of the sperm nuclei is highly compacted and the brightly stained chromocenters are located adjacent to each other (Fig. 2A). Notably, the single-celled pollen produced by *cdka;1^+/−^ fbl17^+/−^* plants displayed a H3K27me1 pattern at anthesis, resembling the changes seen in the vegetative nucleus in the wild type, including the H3K27me1 single spot (Fig. 2B).

A striking characteristics of the differentiation of the vegetative cell is the de-repression of transposable elements (TEs) (Slotkin et al., 2009; Calarco et al., 2012; Ibarra et al., 2012). This de-repression is thought to serve as a source of siRNAs, which accumulate in the sperm cell and drive post-transcriptional silencing of TEs (Slotkin et al., 2009; Martínez et al., 2016). Given that TE activation is a source of genomic instability, we expected that transposons would not be activated before the actual gametes are formed. However, we found that the enhancer trap line ET13889, which resides in AtLine3 (Olmedo-Monfil et al., 2010), showed release of transcriptional repression in single-celled mutant pollen when introgressed into *cdka;1^+/−^ fbl17^+/−^* plants (Fig. S2B, Fig. 2C). Thus, transposons become activated independently of the formation of sperm cells during pollen development.

### Single-celled pollen germinates and targets the ovule

Finally, we asked whether the sperm cells are needed for pollen tube growth and guidance. Previously, it has been shown that colchicine-treated single-cell pollen grains could germinate and grew a pollen tube *in vitro* (Eady et al., 1995). Matching these results, we also found that the *cdka;1^+/−^ fbl17^+/^* single-cell pollen could germinate a pollen tube (Fig. S5). However, whether such a single-cell pollen tube can actually reach an ovule was not clear. To address this question, we attempted to follow pollen tube growth and fertilization using live-cell imaging.

To overcome the limitations of the low rate of single-celled pollen produced in *cdka;1^+/−^ fbl17^+/^*, we decided to use the double mutant combination of *fbl17* with a mutant of *E2FA*. The resulting proportion of single-celled-pollen in this mutant combination is 25% compared with 10% or less in *cdka;1^+/−^ fbl17^+/^* (Zhao et al., 2012) (Fig. S1). To validate whether this single-celled pollen also has vegetative fate, we first analyzed its chromatin state by immunostaining of H3K27me1. This revealed a pattern typical for vegetative cells, similar to the one seen in *cdka;1 fbl17* single-celled pollen (Fig. S6, Fig. 2B). Moreover, we also found that transposon repression of the enhancer trap line ET11075, which resides in an Athila3 transposon (Olmedo-Monfil et al., 2010), was released in the single-celled pollen of *e2fa^−/−^ fbl17^+/−^* plants (Fig. S2B, Fig. S7).

In addition, we analyzed cell fate by germ and vegetative cell fate reporters. To achieve this and as a preparatory measure for subsequent live-cell imaging, we used the reporter system *FB037* (Borges et al., 2012), which contains both *PRO_HTR10_:HTR10:eGFP* and *PRO_ACT11_:H2B:mRFP* (Fig. S2A,B) (Okada et al., 2005; Rotman et al., 2005; Ingouff et al., 2007). Analysis of FB037 in pollen of *e2fa^−/−^ fbl17^+/−^* plants confirmed that the single-celled pollen nucleus has a vegetative fate as the vegetative reporter, but not the generative reporter, was active (Fig. S8). Taken together, this second genetic combination leading to uni-cellular pollen provides independent evidence that the vegetative cell is the default state during pollen development.

**Fig. 3.**
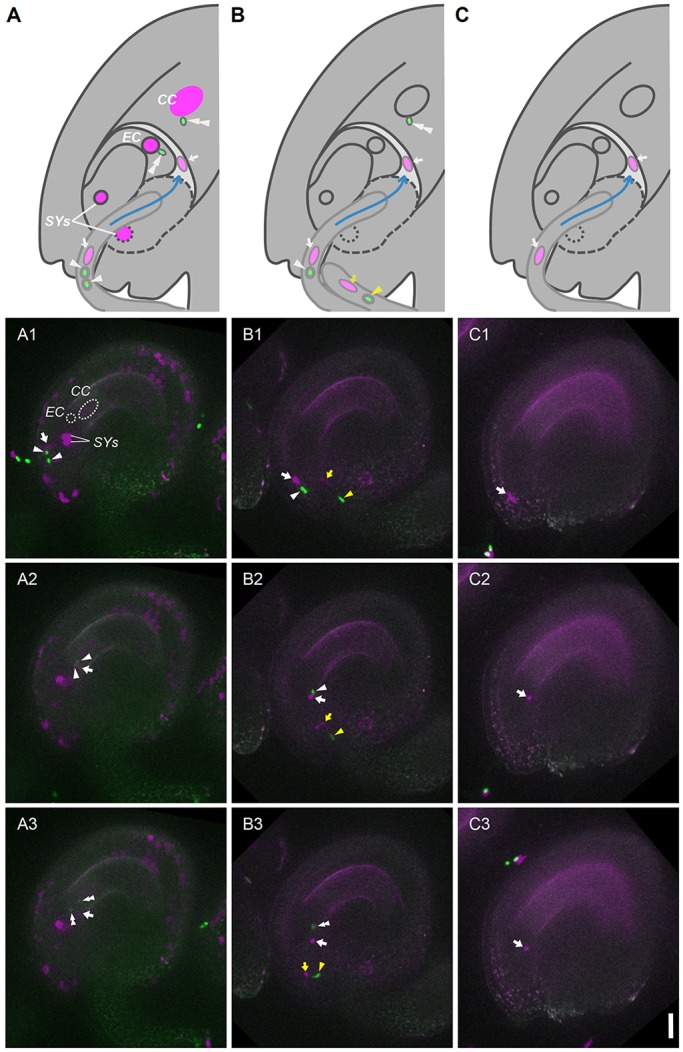
**Fertilization by singled-celled pollen.** (A-C) Summary sketches of the fertilization process shown in the columns below. Numbers from 1 to 3 represent specific fertilization process time points: 1, pollen tube entry into the ovule; 2, post pollen tube discharge; 3, double fertilization. Arrowheads indicate sperm cell nuclei moving towards the egg cell and the central cell; double arrowheads indicate a sperm cell nuclei fertilizing egg and central cell; arrows indicate the vegetative nucleus. EC, egg cell; CC, central cell; SYs, synergid cells. Scale bar: 20 µm. (A) Fertilization of a *PRO_RPS5A_:H2B:tdTomato*-expressing wild-type ovule with a tri-cellular wild-type pollen tube possessing *FB037* marker genes. The RFP-labeled vegetative nucleus and the two GFP-labeled sperm nuclei are visible. (B) Fertilization of a *PRO_RPS5A_:H2B:tdTomato-*expressing ovule with a two-celled (single sperm) *e2fa^−^*^/−^ *fbl17^+^*^/−^ mutant pollen tube possessing *FB037* markers. An RFP-labeled vegetative nucleus and a single GFP-labeled sperm cell nucleus were observed in both the first and second pollen tube approaching (white and yellow arrow and arrowheads). Only one fertilization event is observed. The rod shape appearance of the nuclei results from their movement during image acquisition. (C) Fertilization of a *PRO_RPS5A_:H2B:tdTomato*-expressing ovule with a single-celled *e2fa^−^*^/−^ *fbl17^+^*^/−^ mutant pollen tube possessing *FB037* markers. Only one RFP-labeled vegetative nucleus (arrow) was observed. No fertilization occurs due to the lack of a male gamete.

To assure faithful monitoring of both markers of *FB037* in the segregating meiotic products of *e2fa^−/−^ fbl17^+/−^* plants, the cell fate reporters of the *FB037* line were brought into a homozygous state. To observe the behavior of the RFP-labeled vegetative nucleus and GFP-labeled sperm cell nuclei, we performed live-cell imaging using a previously established semi-*in vivo* fertilization assay (Fig. 3; Movies 1-3) (Palanivelu and Preuss, 2006; Hamamura et al., 2011).

When tri-cellular wild-type pollen was used as a male donor, pollen tubes were attracted to and entered the egg-apparatus of an ovule (Fig. 3A). The vegetative nucleus and the two sperm cell nuclei moved backwards and forwards together in a pollen tube that had entered an ovule (Fig. 3A1; Movie 1). The three nuclei were then suddenly discharged into the ovule (Fig. 3A2; Movie 1), as described previously (Hamamura et al., 2011). The RFP-labeled vegetative nucleus stayed at the released position, while the two GFP-labeled sperm cell nuclei began to move towards the nuclei of the fertilization targets, the egg cell and the central cell, indicating successful double fertilization (Fig. 3A3; Movie 1).

Next, we used the *e2fa^−/−^ fbl17^+^*^/−^ double mutant with the same *FB037* reporter system as a pollen donor. As in the case of wild-type pollen tubes, the tri-cellular, wild-type like pollen tube penetrated into the ovule and discharged its contents, including two sperm cells, fertilizing the two female gametes (*n*=5; data not shown). We also observed that the two-celled pollen tubes of the mutant grew normally and discharged their contents, including a single sperm cell, which fertilized the egg cell or the central cell (*n*=2; Fig. 3B; Movie 2). This suggested that the function of not only the vegetative cell but also the sperm cell is normal for fertilization in the two-celled pollen, consistent with earlier data (Nowack et al., 2006).

Remarkably, the single-celled pollen tube with only a RFP-labeled vegetative nucleus also elongated normally, entered into the ovule and discharged its contents, including the vegetative nucleus (*n*=4; Fig. 3C; Movie 3). Consequently, we concluded that the single-celled pollen of the *e2fa^−/−^ fbl17^+^*^/−^ double mutant generated a pollen tube with normal function in ovule targeting and discharge.

**Fig. 4.**
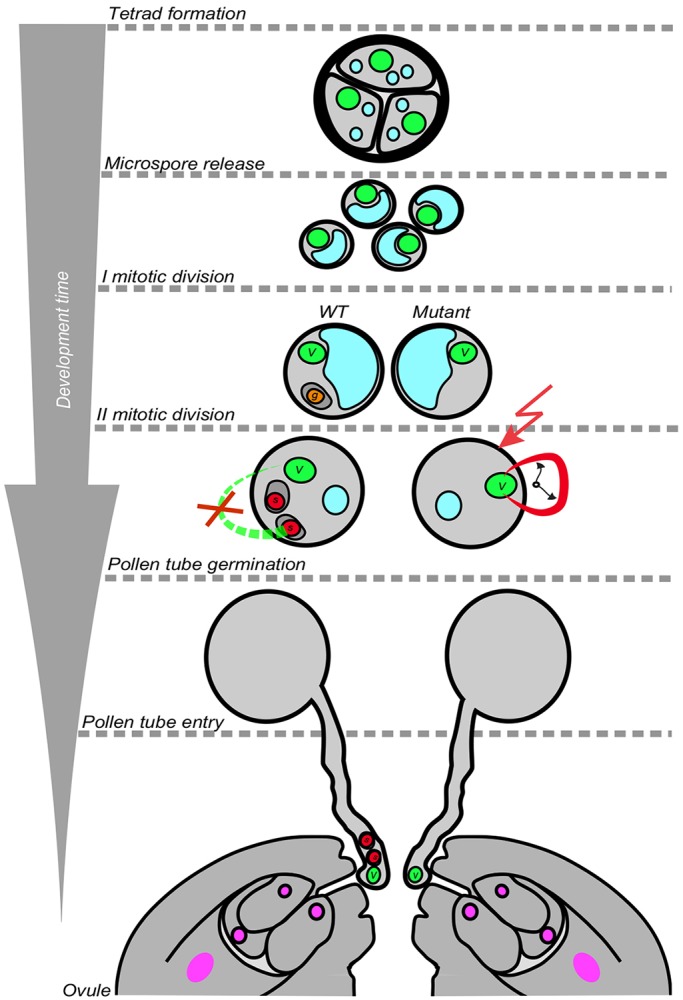
**Model of pollen differentiation.** At the end of meiosis, uni-cellular microspores are released from the tetrad. After the first unequal mitotic division, a vegetative cell (v) and a generative cell (g) are formed (left, wild type). Without this division (right, mutant), the single cell in the pollen develops vegetative characteristics, including the release of transposon repression. Differentiation of the vegetative cell is independent of the presence of the sperm cell(s) formed at PMII. Transcriptional release of transposons in the vegetative nucleus is therefore either an autonomous process or due to external cues (indicated by the clock hands and lightning bolt, respectively). The single-celled pollen accomplish pollen tube guidance and discharge to the ovule as in the wild type. Blue, vacuole; green, microspore and vegetative nuclei; orange, generative cell nucleus; red, sperm nuclei; magenta, female gametophyte nuclei.

Taken together, our work demonstrates that the default state of microspores is a vegetative fate (Fig. 4). This is consistent with earlier work using the spindle poison colchicine to block PMI or using mutants that interfere with pollen cytokinesis, such as gem1 (Eady et al., 1995; Park et al., 1998). If the formative division is absent, differentiation of the vegetative cell proceeds independently of the presence of gametes or their precursors, and either progresses autonomously (i.e. promoted by an internal pace maker) or is driven by an external signal, e.g. from the maturation of cells surrounding the microspore in the anther (Fig. 4). Remarkably, the vegetative cell is sufficient for pollen tube guidance and discharge, indicating that the male gamete is just a cargo for DNA (Fig. 4). The same conclusion was recently drawn from independent experimental work using basic helix-loop-helix (bHLH) transcription factor gene double mutants – *DEFECTIVE REGION OF POLLEN 1* (*DROP1*), also known as *LJ-RHL1-LIKE* (*LRL1*) and *DROP2* (*LRL2*) – which also develop ∼40% single-celled pollen at anthesis (Zhang et al., 2017).

## MATERIALS AND METHODS

### Growth conditions and plant materials

*Arabidopsis thaliana* (L.) lines derived from ecotype Col-0 (Table S1) were first grown on half Murashige Skoog medium agar plates with addition of 0.5% sucrose in long-day conditions (16 h day/8 h night) at 18°C and 21°C during the day and at 18°C during the night, and then transferred on soil and grown in long-day conditions (12/12 h day/night) with the same temperature settings. All genotypes were determined by polymerase chain reaction (PCR) with the primers indicated in Table S2. Phenotypic analysis was always carried out with pollen from at least three different plants.

All mutants and reporter lines used in this study have been described previously if not indicated otherwise (Table S1). For mutants in *CDKA;1* (AT3G48750), the previously described allele *cdka;1-1* was used (Nowack et al., 2006); for mutants in *FBL17* (At3g54650), the allele *fbl17-1* (GABI-170E02) was used (Gusti et al., 2009); and for E2FA (At2g36010), the allele *e2fa* (GK_348E09) was used (Zhao et al., 2012). The double mutants *cdka;1 fbl17* and *e2fa fbl17* have been previously presented (Zhao et al., 2012). The enhancer trap lines ET13889 and ET11075 have been described previously (Slotkin et al., 2009).

The male gamete lineage markers *PRO_HTR10_:HTR10:RFP* and *PRO_HTR12_:HTR12:GFP* have been described by Fang et al. and Ingouff et al., respectively (Fang and Spector, 2005; Ingouff et al., 2007). The vegetative and gamete lineage reporter system *FB037* containing both *PRO_HTR10_:HTR10:eGFP* and *PRO_ACT11_:H2B:mRFP* has been described previously (Borges et al., 2012). The transgenic *A. thaliana* plants possessing the *PRO_RPS5A_:H2B:tdTomato* used to visualize female gametophytic cell nuclei have been described previously (Adachi et al., 2011).

Counting of pollen and analysis of cell fate reporters were carried out at anthesis, i.e. when pollen is released from open flowers. The *PRO_CSLS4_:GUS* reporter construct was generated using PCR with primers *spCSLD4_attb1* and *aspCSLD4_attb1* (Table S2), recombined into *pDONRZeo* (Invitrogen) and further recombined to the binary vector *pMDC162* (Curtis and Grossniklaus, 2003). The destination vector was transformed into *Agrobacterium* strain C58 pGV2260 and further brought into *Arabidopsis Col-0* using the floral dip method (Clough and Bent, 1998) as described previously (Bjerkan et al., 2012).

### Histology

For inspection of pollen phenotypes, anthers of different stages were mounted directly in staining buffer [50 mM NaPO_4_ buffer (pH 7.2); 5% DMSO; 0.01% Tween 20; H_2_O] containing 2 µg/ml DAPI as described previously (Grini et al., 1999). Anthers were squashed by gently tapping on the cover slip, and preparations were sealed with nail polish and incubated in the dark at 4°C for 3-24 h before inspection. Rare cases of unstained pollen were omitted from statistics.

GUS assays were performed according to Grini et al. (2002) and incubated in the dark for up to 2 days. For the combined inspection of reporter lines and pollen phenotypes, marker lines were incubated using the DAPI protocol described above. For GUS staining, whole flowers were incubated in the previously described GUS staining solution, applying gentle vacuum (15 mPa) for 15-30 min before overnight incubation. GUS-treated single anthers were mounted in DAPI solution as described above.

### *In vitro* pollen tube germination assays

Mature pollen grains were placed on medium prepared as described previously (Li et al., 1999) with some minor modifications [18% sucrose, 0.01% boric acid, 1 mM CaCl_2_, 1 mM Ca(NO_3_)_2_, 1 mM MgSO_4_, 0.5% Noble agar (Difco) (pH 7.0)]. Pollen were germinated at 20-22°C for 6-10 h in humid conditions before they were examined using a light microscope.

### Semi-*in vivo* fertilization assay

A semi-*in vivo* fertilization assay was conducted as described previously (Palanivelu and Preuss, 2006; Hamamura et al., 2011) with slight modifications; pollen germination medium [0.01% (w/v) boric acid, 5 mM CaCl_2_, 5 mM KCl, 1 mM MgSO_4_, and 10% (w/v) sucrose], adjusted to pH 7.5 with KOH and solidified with 1% (w/v) low melting temperature agarose (NuSieve GTG Agarose; Lonza) (Boavida and McCormick, 2007). Time-lapse images were taken about 3 h after pollination to trace the pollen tubes, ovule targeting and discharge of the pollen tube contents.

### Immunostaining procedure

The procedures used for immunostaining were modified from Lauber et al. (Lauber et al., 1997). Whole flowers were collected and submerged in 3.6% formaldehyde in 1×PBS buffer, vacuum-infiltrated at 8 mm Hg at room temperature for 60 min and washed twice for 10 min in 1×PBS followed by a dehydration series in ethanol in 1×PBS (10%, 25%, 40% and 55% ethanol in deionized H_2_O; 15 min each step. For storage at 4°C, the flowers were transferred to 70% ethanol in deionized H_2_O. Before antibody staining, the flowers were rehydrated using the reverse of the series above. A single anther of one flower was placed on a polylysine-coated object glass in a 10 µl droplet of 1×PBS, covered with a cover slip and the specimen was carefully squeezed to release pollen from the anthers. The slide was immediately submerged in liquid nitrogen for 15 s and the cover slip was blasted off using a razor blade. The slide was dried at 37°C until no liquid remained, the sample was circled in with microscopy grease and washed for 5 min by applying a drop of 40 µl 1×PBS. A solution of Drisilase (from Basidomycetes, Sigma) was prepared by mixing 0.02 g per 100 µl of 1×PBS and shortly centrifuged until a pellet was formed. At least 40 µl of the supernatant was added to the sample and incubated at 37°C for 3 h in a humid chamber. For the early developmental stages of pollen, the Drisilase solution was refreshed after 2 h and incubated for an additional 2 h (4 h in total). The specimen was washed three times for 10 min with 40 µl of 1×PBS in a humid chamber at room temperature. At least 40 µl of a mixture of 1% of Triton X-100 and 10% DMSO in 1×PBS was added to the sample and incubated for 1 h at 37°C in a humid chamber. The specimen was washed again three times for 10 min with 40 µl of 1×PBS in a humid chamber at room temperature. The sample was blocked in 40 µl of 1% BSA in 1×PBS and incubated in a humid chamber at 4°C overnight. Primary antibody (40 µl) (Table S3) was applied at a concentration of 1:100 in 1% BSA in 1×PBS and incubated in a humid chamber at 4°C for 12 h. The primary antibody solution was removed from the slide and rinsed with 40 µl 1×PBS followed by washing twice for 10 and 15 min with 40 µl 1×PBS in a humid chamber at room temperature and finally incubated overnight with 40 µl 1×PBS in a humid chamber at 4°C. Secondary antibody (40 µl) (Table S3) was applied and incubated in a humid chamber at 4°C for 12 h.

The secondary antibody solution was removed from the slide, the slide was washed twice for 10 and 15 min with 40 µl 1×PBS in a humid chamber at room temperature, and finally incubated overnight with 40 µl 1×PBS in a humid chamber at 4°C. The microscopy grease and all the remaining liquid were removed after the final washing step and 15 μl DAPI solution (in 0.5×PBS, 5% DMSO, 0,01% Tween) was added to the specimen. The cover slip was gently applied on top of the specimen. The coverslip was sealed with non-fluorescent nail polish and incubated at 4°C overnight before inspection.

### Microscopy

Unless otherwise indicated, all images were generated using an AxioCam HRc mounted on a Zeiss Axioplan 2 imaging microscope with the software AxioVision version 4.8; with an Axio Imager.Z1 equipped with an AxioCamMR3; or with a confocal microscope OLYMPUS BX61Wi with the software OLYMPUS FLUOVIEW version 3.1. Microscopic images were processed using Adobe Photoshop and Illustrator software.

For live-cell imaging of semi-*in vivo* fertilization, the following microscopy settings were used as described previously (Hamamura et al., 2014; Gooh et al., 2015). Confocal images were acquired using an inverted microscope IX-83 (Olympus) equipped with a disk-scan confocal system (CSU-W1; Yokogawa Electric). A silicone oil immersion objective lens, UPLSAPO60XS (Olympus), mounted on a Piezo z-drive (P-721; Physik Instrumente) was used. Time-lapse and *z*-stack images were acquired every 5-10 min in seven planes (3 μm intervals). The exposure time of 488 nm laser was 250-300 ms for eGFP, and of 561 nm laser was 50-200 ms for mRFP and tdTomato. Images were processed by Metamorph version 7.8.4.0 (Universal Imaging) to display maximum-intensity projection images and to add pseudo-colors. The images and movies were edited by MacBiophotonics ImageJ software (http://www.macbiophotonics.ca/).

## Supplementary Material

Supplementary information

Supplementary information
