# Supplementary material for: Pollen differentiation as well as pollen tube guidance and discharge are independent of the presence of gametes
Source: Development. 2018 Jan 1;145(1):dev152645. doi: 10.1242/dev.152645 (PMC5825867; doi:10.1242/dev.152645)
Supplement: Supplementary information [file develop-145-152645-s1.pdf]

Supplementary Figures

S1

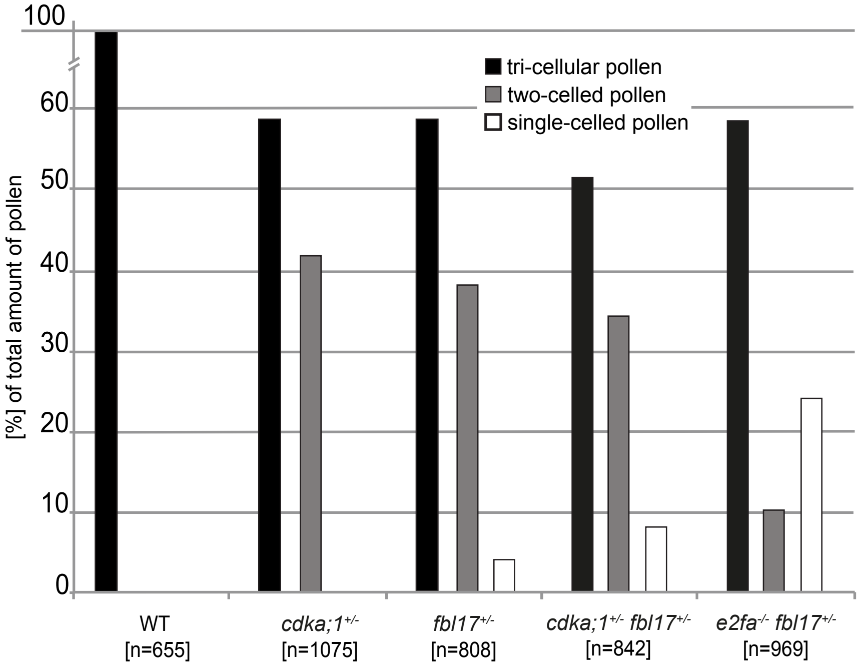

Supplementary figure 1. Frequencies of pollen phenotypes

Frequencies of mutant, i.e. single-celled and two-celled, pollen versus wild-type and wild-type like tri-cellular pollen of the mutants used in this study in comparison with the wildtype.

Development • Supplementary information

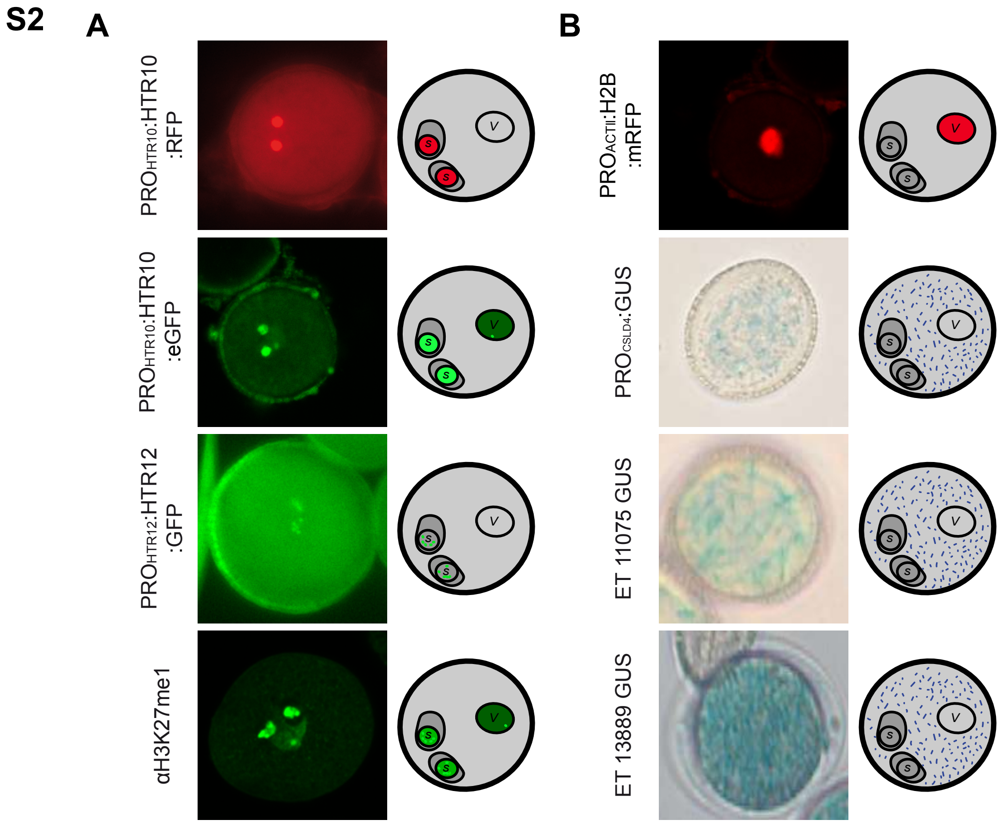

Supplementary figure 2. Overview over cell fate markers used in this study

A. Sperm cell fate markers. Left column, micrograph and the right column, model of the respective marker. Please note that the sperm cell fate marker HTR10 is also weakly expressed in the vegetative cell. However, given the strong difference in expression levels between the vegetative cell and the sperm cells, it is a suitable marker to discriminate between these two cell types. Similarly, the chromatin mark H3K27me1 is also found in the vegetative cell. However, in contrast to the sperm cells where this mark is associated with chromocenters, the signal is very dispersed in the nucleus of the vegetative with the exception of one distinct dot. Hence, this characteristic pattern can also be used to distinguish between these two cell types.

B. Vegetative cell fate markers. Left column, micrograph and the right column, model of the respective marker.

Vegetative cells are depicted with light grey whereas sperm cells are shown in dark grey. v: nucleus of the vegetative cell. s: nucleus of the sperm cell.

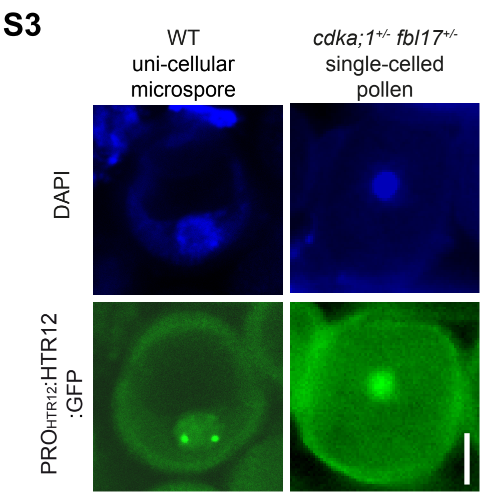

Supplementary figure 3. HTR12 expression in uni-cellular microspores

Fluorescent micrographs of uni-cellular microspores containing a *PRO$_{HTR12}$HTR12:GFP* reporter construct. HTR12:GFP specifically marks the centromeres of nearly all sperm cell nuclei in the mature tri-cellular pollen grain. In contrast, it is absent in *cdka;1 fbl17* mutant pollen, see Fig. 1A. However, HTR12:GFP is expressed in the uni-cellular microspores of both the wildtype and *cdka;1 fbl17* mutants indicating that the single celled pollen of *cdka;1$^{+/-}$ fbl17$^{+/-}$* mutants is not an arrested microspore. Scale bar is 5 µm.

S4

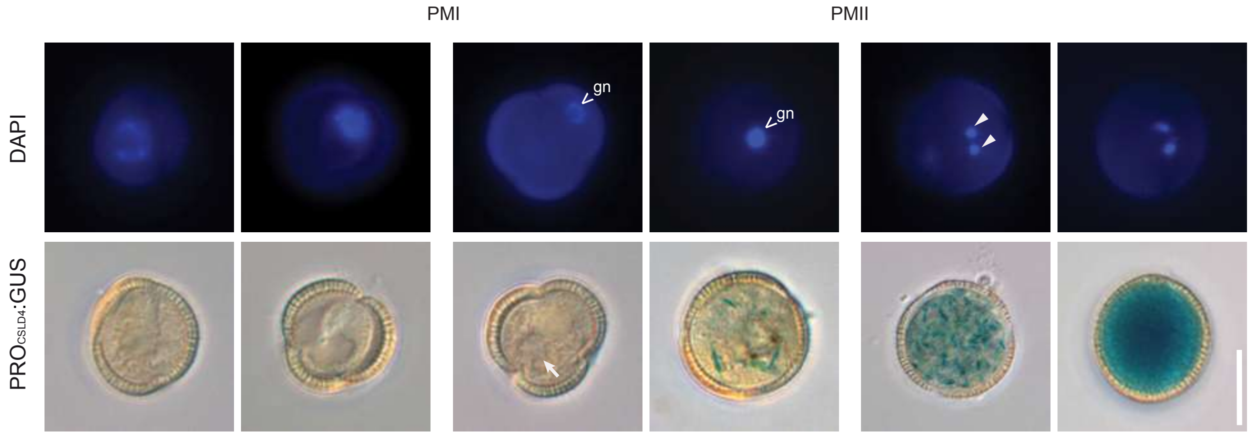

Supplementary figure 4. Expression analysis of CSLD4 during pollen development

Fluorescent- and light micrographs of developmental stages of wild type pollen containing a $PRO_{CSLD4}GUS$ vegetative cell reporter construct. Upper rows DAPI stained pollen, lower rows bright field signal of GUS staining of the same pollen. In early developmental stages up to PMI, no $PRO_{CSLD4}GUS$ activity could be detected. Shortly after the release of the generative nuclei from the pollen wall, $PRO_{CSLD4}GUS$ expression can be detected in the vegetative cell cytoplasm. The GUS signal is getting more pronounced just after PMII and accumulates in the vegetative cell of the mature pollen. Arrowheads indicate sperm cell nuclei, arrows designate vegetative nuclei, gn indicate generative nucleus. Scale bar is 5 µm.

S5

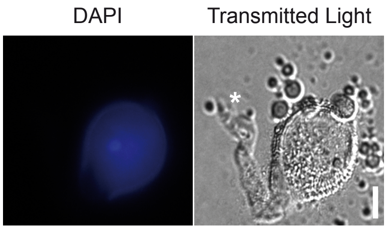

Supplementary figure 5. Pollen germination assay of single-celled pollen

Fluorescent- and light micrographs of *in vitro* germinated single-celled pollen from *cdka;1* $^{+/-}$ *fbl17* $^{+/-}$. The first column shows DAPI staining with only one nucleus visible in the germinating pollen grain. In the second column the germinating pollen tube from the same pollen is marked with an asterisk. Scale bar is 5 µm.

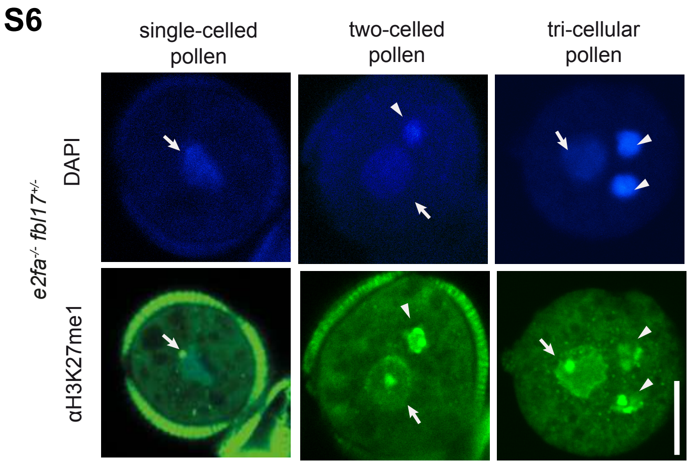

Supplementary figure 6. Chromatin differentiation in single-cell pollen of *e2fa* $^{-/-}$ *fbl17* $^{+/-}$ plants

Fluorescent micrographs of pollen from *e2fa* $^{-/-}$ *fbl17* $^{+/-}$ plants. The upper row shows DAPI staining and the lower row whole mount immunodetection of H3K27me1 in the same pollen. In the leftmost column the single-celled pollen nucleus is showing the same H3K27me1 pattern as the vegetative nucleus of the mature WT pollen (Fig. 2A). A bright spot is visible as well as a weaker signal distributed over the whole nucleus. The two-celled pollen in the middle column has a vegetative nucleus with the same pattern as the vegetative nucleus of a WT tri-cellular pollen and the generative nucleus has the same pattern as the sperm cells of the tri-cellular WT pollen. In the tri-cellular pollen in the rightmost column the same H3K27me1 distribution pattern as in tri-cellular WT pollen is observed. Scale bar is 5 μm.

Development • Supplementary information

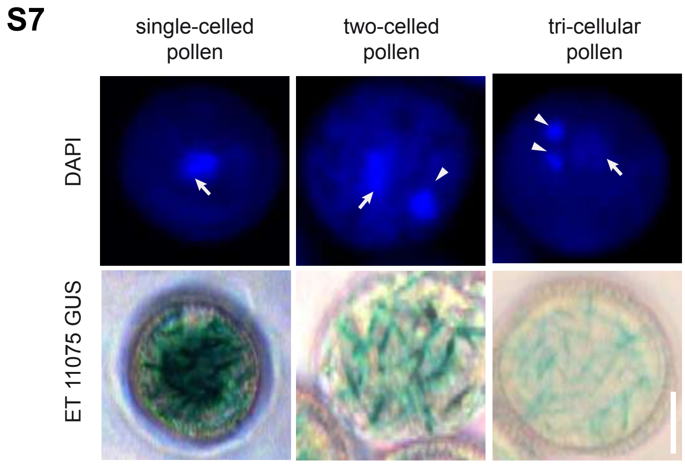

Supplementary figure 7. Activation of transposon expression in single-celled pollen of *e2fa*$^{-/-}$ *fbl17*$^{+/-}$ plants

Fluorescent- and light micrographs of single-celled, two-celled and tri-cellular pollen of *e2fa*$^{+/-}$ *fbl17*$^{+/-}$ plants expressing the Ds transposon Enhancer trap line ET11075 GUS reporter. All three pollen types of *e2fa*$^{+/-}$ *fbl17*$^{+/-}$ plants express the GUS reporter. In the upper row DAPI staining is shown, the lower show GUS staining. The arrowheads indicate the sperm cell, arrows indicate the vegetative nucleus. Scale bar is 10 µm.

Development • Supplementary information

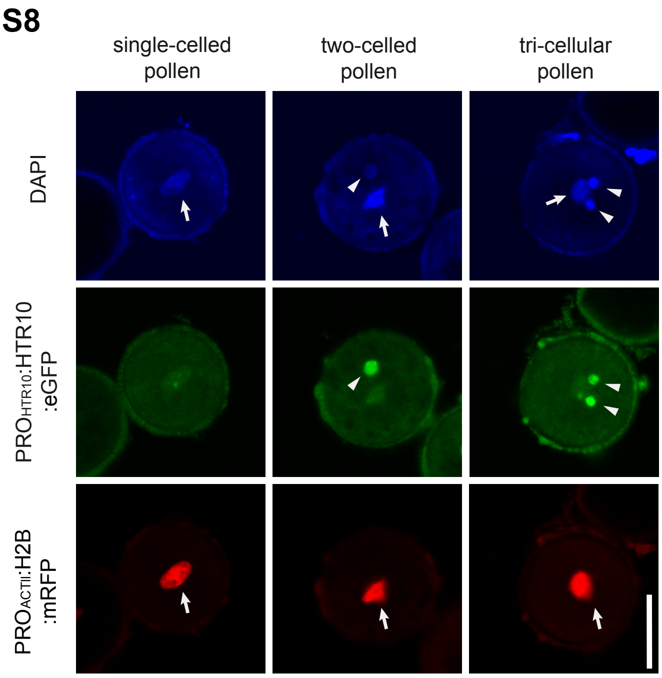

Supplementary figure 8. Cell fate analysis of single-celled pollen of *e2fa$^{-/-}$ fbl17$^{+/-}$* plants

Fluorescent micrographs of pollen from *e2fa$^{-/-}$ fbl17$^{+/-}$* plants carrying *FB037* reporters *PRO$_{HTR10}$:HTR10:eGFP* and *PRO$_{ACT11}$:H2B:mRFP*. The columns represent, DAPI staining of all nuclei in the pollen grain, *PRO$_{HTR10}$:HTR10:eGFP* marking the generative nuclei and *PRO$_{ACT11}$:H2B:mRFP* marking the vegetative nucleus. The first, second and third row show panels with single celled pollen, two celled pollen and tri-cellular pollen, respectively. Arrowheads indicate sperm cell nuclei, arrows designate vegetative nuclei. Scale bar is 10 µm.

Development • Supplementary information

Supplementary Tables Glöckle and Urban et al.

Supplementary Table 1

Line	Background ecotype	Source
ET13889	Ler	Slotkin et al.,2009
ET11075	Ler	Slotkin et al.,2009
cdka;1 (SALK_106809)	Col-0	Alonso et al. 2003
fbl17 (GABI-170E02)	Col-0	Kleinboelting et. al. 2012
e2fa (GK-348E09)	Col-0	Kleinboelting et. al. 2012
PRO$_{HTR10}$HTR10-RFP	Col-0	Ingouff et al., 2007
PRO$_{HTR12}$HTR12-GFP	Col-0	Fang et al., 2005
PRO$_{CSLD4}$GUS	Col-0	This report
FB037 reporter system containing PRO$_{HTR10}$:HTR10:eGFP and PRO$_{ACTII}$:H2B-RFP	Col-0	Borges et al., 2012
ProRPS5A:H2B-tdTomato	Col-0	Adachi et al., 2011

Supplementary Table 2

TDNA genotyping primers			
108	CDKA;1 (Salk)	5'-TTTGGCTGGCTGCATTCCTTA-3'	CDKA;1
S1		5'-GCGTGGACCGCTTGCTGCAACTCTCTCAGG-3'	T-DNA left border
8409	FBL17 (GK)	5'-ATATTGACCATCATACTCATTGC-3'	T-DNA left border
FBLr		5'-CAGATGTTCAAGGGATTACC-3'	FBL17
GABI	E2Fa (GK)	5'-CCCATTTGGACGTGAATGTAGACAC-3'	T-DNA left border
B256		5'-TTCCAGGTCTGTCTTTCCTATTTC-3'	E2Fa
WT genotyping primers			
108	CDKA;1 (Salk)	5'-TTTGGCTGGCTGCATTCCTTA-3'	CDKA;1
14		5'-TGTACAAGCGAATAAAGACATTTGA-3'	CDKA;1
FBLf	FBL17 (GK)	5'-GGTGGCATTCAATTTGCTAC-3'	FBL17
FBLr		5'-CAGATGTTCAAGGGATTACC-3'	FBL17
B256	E2Fa (GK)	5'-TTCCAGGTCTGTCTTTCCTATTTC-3'	E2Fa
B257		5'-ATTCCTCCTACTTGCTCTTGC-3'	E2Fa
pro:CSLD4 cloning primers			
aspCSLD4_attb1	GGGGACAAGTTTGTACAAAAAAGCAGGCTGTTCTGCGACGACGACATCTAACG		
spCSLD4_attb1	GGGGACCACTTTGTACAAGAAAGCTGGGTCAAACAAAGGAGCTTGGTTTCTGC		

Development • Supplementary information

Supplementary Table 3

1st / 2nd	Antibody	Company Lot	Type
1st	α-H3K27me1	Millipore 07-448 Lot:	rabbit polyclonal
2nd	AlexaFluor ® 488	Invitrogen Lot: 94C2-1	goat anti rabbit IgG (H+L)
2nd	AlexaFluor ® 555	Invitrogen Lot: 52944A	goat anti rabbit IgG (H+L)

Supplementary movies

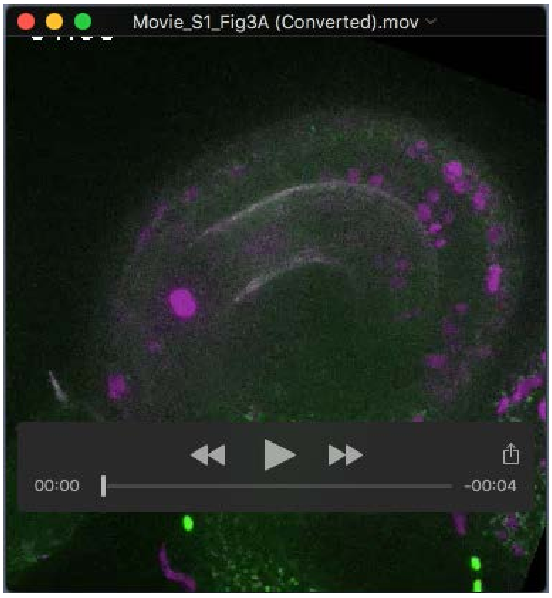

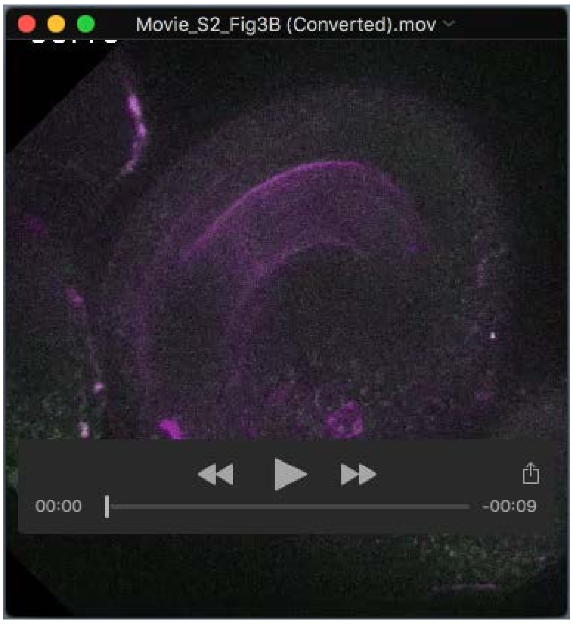

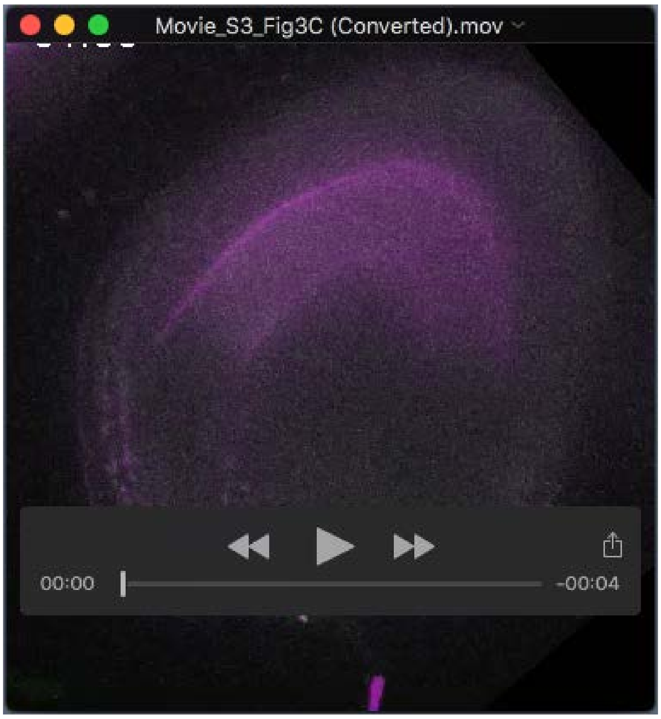

Supplementary movies S1-S3. Live-cell imaging of the fertilization process of one-celled pollen.

The movies S1–3 correspond to Fig. 3A–C, respectively. Time indicates elapsed time; 00:00 indicates the last frame just before pollen tube discharge.

Development • Supplementary information
